# A Human-Centered Platform for HIV Infection Reduction in New York: Development and Usage Analysis of the Ending the Epidemic (ETE) Dashboard

**DOI:** 10.2196/publichealth.8312

**Published:** 2017-12-11

**Authors:** Ashish Joshi, Chioma Amadi, Benjamin Katz, Sarah Kulkarni, Denis Nash

**Affiliations:** ^1^ Institute for Implementation Science in Population Health Graduate School of Public Health and Health Policy City University of New York New York, NY United States

**Keywords:** geo-visualization, ETE dashboard, HIV/AIDS, human centered design

## Abstract

**Background:**

Dashboards have been increasingly used in clinic-based interventions, such as clinical performance improvement and monitoring risk of hospital readmissions, and are now gaining traction in population-based interventions, especially in disease assessment.

**Objective:**

We describe the design, development, and usage analysis of a geovisualization dashboard, the Ending the Epidemic (ETE) Dashboard. The ETE dashboard is a tool developed to track New York’s progress towards achieving the goal of its ETE Initiative, to reduce new HIV infections from 3000 per year to 750 per year by the end of 2020.

**Methods:**

The ETE dashboard was adapted from an existing human-centered geovisualization platform, SanaViz, an Internet-enabled, interactive app incorporating principles of human-centered design and cognitive fit theory to enhance visual exploration of population health data. Usage evaluation of the ETE geovisualization dashboard was conducted using Google Analytics over a 4-week period from March 19 to April 18, 2016. The aim was to monitor user activity and analyze traffic on the ETE dashboard using evidence-based metrics that can provide adequate feedback to enhance its utilization. Usage was characterized based on three metrics: (1) number of unique visits to each page, (2) average time on each page in seconds, and (3) page bounce rate (ie, percentage of visits where user left the site immediately after viewing just a single page). Further analysis was also conducted by cross-tabulating specific usage metrics.

**Results:**

Of 860 sessions, 324 sessions were initiated by unique users (37.7%). The most common acquisition channels included direct source (353/860, 41.0%); followed by referral traffic (340/860, 39.5%) and organic search (134/860, 15.5%). Usage statistics indicate that for the 860 sessions initiated by both new and returning users, the average viewing time was 8 minutes, 51 seconds, and the bounce rate was 46%. These statistics reflect positive results given that prior literature estimates an average session duration of 10-20 seconds and a bounce rate of 40-60% for most websites. Key findings from our study showed that the highest bounce rates were seen for the “About” page of the ETE website (65%), which describes the ETE initiative. The lowest bounce rates were seen for the ETE blog page (17%), consistent with prior research on website usage metrics that indicate that blog pages are often the most frequently viewed pages on Web portals.

**Conclusions:**

Our findings reveal the potential of Google Analytics as a tool to enhance user traffic and performance of the ETE geovisualization platform by using feedback from regular monitoring of key parameters including page bounce rates and average time on page. It also identifies the need for a follow-up usability assessment of the system.

## Introduction

Global population growth has been accompanied by a corresponding expansion of health datasets. The aggregation of these health datasets from diverse sources including the pharmaceutical, medical, and health insurance sectors is commonly referred to as big data. The relevance of big data in health care has been exemplified by its use in assessing hospital readmission patterns, mining of medical data to identify most effective treatments for health conditions, and identifying innovative methods to reduce patient costs [1]. Generating such aggregate data is facilitated by advances in technology, as recent trends indicate a rise in innovative tools to foster improved dissemination and use of health data in decision making [1]. Integration of health data has been defined by the Association for Behavioral Health and Wellness as “whole person care that focuses on overall health; creates partnerships across all aspects of health; and is facilitated by a variety of clinical, structural, financial arrangements and community supports that remove barriers between physical and behavioral health care” [2]. Health data integration offers immense potential to foster efficiency, improve quality of care, increase patient satisfaction, and ultimately promote population health outcomes [3]. Integrated technology platforms both benefit from and foster clinical integration, which leads to increased collaboration of health care providers, reduced waste of health care resources, improved care coordination, patient-centered communication, enhanced pharmaceutical management, and improved health data systems development [3].

Dimensions of public health data include spatial (ie, geographic), temporal (ie, time), and attribute (ie, context), which provide valuable insight into tackling population-level health issues and support effective decision making. Spatial components refer to location-related features such as region or country [4]. The temporal component refers to timing of the occurrence or conditions and is useful in describing trends over time [5], while attributes refer to the specific characteristic of interest. These factors combined inform stakeholders in monitoring, disseminating, and improving population-level outcomes. Visualization tools provide an immense capacity to examine the various dimensions of public health data including spatial, temporal, and other attributes, as well as intuitive exploration of the relationships between the various elements beyond the capacity of traditional statistical analysis [6]. Dashboards are visualization tools that present aggregated data graphically, thereby streamlining cognitive effort in assessing decision alternatives while providing the users with the ability to self-select the desired information for display and to explore [7,8]. Empirical studies have identified numerous uses and features of dashboards including dissemination of integrated data to a broad audience, presentation of coding systems to alert users of essential events or changes in metrics [7], efficient analytical mechanisms to provide rapid feedback on key metrics and outcome measures [7], comprehensive summaries of multiple information sources in a single view [9], systems for early detection of adverse events, facilitation of informed decision making in clinical and community settings [7-9], and user-centered and interactive features that allow manipulation of data displays based on desired outcomes [9].

Dashboards have been increasingly used in clinic-based interventions, such as clinical performance improvement and monitoring risk of hospital readmissions, and are gaining traction in population-based interventions, especially in disease assessment [10]. The efficiency of dashboards are exemplified in their description as a single platform incorporating multiple functions including data collection tools, data aggregation, data analysis, mapping/geographic information system technology, and data visualization [7]; or as a combination of one or more of these platforms [11]. Dashboards have been described as conceptual design frameworks in the existing literature based on their applications in health improvement, design features, evaluation reports, and implementation challenges [9]. A commonality in the features of dashboards across these empirical studies is the flexible architecture of dashboards to incorporate various functionalities [8]. Data visualization has also been identified as a key component of dashboards, largely attributable to cognitive theories that underscore the importance of using graphs in examining relationships and recognizing patterns to facilitate real-time data dissemination and decision making, which is preferable to tables and static reports [12].

Prior research has utilized a variety of Google Analytics metrics in website performance and informing their design and development. They include metrics for (1) evaluating and improving content (ie, search engine, key words, top entry and exit pages, page viewing time, and referrers), (2) enhancing navigation (ie, search key words, error pages, path analysis), (3) evaluating accessibility (ie, search key words, search engines, entry pages, referrer), and (4) informing site design (ie, browser usage and platform statistics) [13], a framework consisting of 20 log-based metrics for enhancing user experience [14]. Our study used a combination of these Google Analytics metrics in evaluating usage of the Ending the Epidemic (ETE) website. The objective of this study is to describe the design, development, and a usage analysis of the ETE geovisualization platform, which was adapted from an existing human-centered framework, SanaViz.

### Challenges in Geovisualization

Geovisualization is a technique that uses mapping tools to represent data insights and visual interactions [15]. Through its capacity to generate geospatial displays of aggregated data, geovisualization provides an ideal and efficient way to generate hypotheses and foster evidence-driven solutions [16]. The use of maps provides a user-friendly approach to view complex data in a simplified way [16]. Maps have the potential to elicit visual thinking and readily understood patterns and relationships between data elements that are key for data exploration, hypothesis generation, and decision making [17]. Despite the immense potential of geovisualization and spatial analysis, their use in population health has been limited [16]. This has been attributed to a lack of sufficient research that provides detailed guidance on design and development of user-friendly geovisualization tools for use in population health [18,19].

Prior research has shown that the design framework of major geovisualization tools and technologies solely utilizes information technology and software engineering principles. These frameworks do not incorporate adequate user input into the initial design phases, but rather they are included after the main functionality and interface requirements have been decided [20,21]. The resulting geovisualization apps are often generic, lack user-friendliness, and fail to meet the range of user-specific needs [18]. The need for human-centered theoretical frameworks is pertinent in the design and implementation of geovisualization tools and technologies to enhance their usage and benefits.

### Role of Human-Centered Approach in the Design of the ETE Dashboard

Understanding user characteristics, needs, and preferences are critical for developing human-centered geovisualization tools and technologies such as the ETE dashboard. Analysis of user demographics such as age and education, as well as prior exposure to and familiarity of spatial visualization and computers play a key role in ensuring optimal utilization of geovisualization apps. The primary feature of user interactivity in geovisualization platforms facilitates control over the display of the data, enables comparisons and layering of different mapping environments, and presents complex data in a format that can be easily appreciated by the user. The format in which visual displays are used to present information will largely influence information processing and knowledge generation. Creating the most effective display format to improve information processing and knowledge generation remains a crucial challenge in most geovisualization platforms. In addition, user tasks and operations need to be clearly defined and collectively evaluated to meet the goals of data exploration, analysis, and knowledge development [21,22].

### Description of the ETE Dashboard

The ETE dashboard is a geovisualization dashboard designed to measure, track, and disseminate actionable information on progress towards achieving the goals of New York State’s ETE Initiative to all stakeholders. The information contained in the dashboard system needs to be accessible and useful to a very wide array of stakeholders ranging from policy makers who often want high-level information to practitioners, community-based organizations, and advocacy organizations that often request more granular and technical information. The ETE geovisualization dashboard was adapted using the SanaViz platform (meaning “health view”) [23]. The dashboard included 2 phases: (1) a static component and (2) an interactive component. The primary subject matter experts who informed the content and interface of the ETE dashboard included the New York State Department of Health AIDS Institute, the New York City Department of Health and Mental Hygiene, the data subcommittee of the New York State Ending the Epidemic Task Force, and the Institute for Implementation Science in Population Health at the City University of New York (CUNY), and the CUNY Graduate School of Public Health and Health Policy.

The ETE geovisualization dashboard provides information about different data sources that have been used to present HIV epidemic-related information in static as well as interactive formats. The information is presented using a combination of maps, charts, and graphs. The dashboard has a series of interactive features including highlights and multiple linkages to facilitate visual exploration of the data across different perspectives. Individuals using the interactive version of the dashboard can filter the variables of interest to present the data in a meaningful format using a combination of maps, graphs, and charts. The dashboard also has a blog that is regularly updated to communicate HIV and acquired immune deficiency syndrome (AIDS) epidemic-related information. The dashboard facilitates data entry, import, export, and integration across different data sources. The Measures tab represents seven broad data realms of HIV/AIDS information including prevention of HIV infections, incidence, testing, new diagnoses and linkage, prevalence and care of HIV/AIDS, AIDS diagnoses, and deaths among people living with HIV/AIDS. Additional features of the dashboard include information on events and news as well as HIV/AIDS-related resources (Multimedia Appendices 1 and 2).

### Technical Architecture and Technologies Used

The ETE geovisualization dashboard is built on open source technologies including hypertext preprocessor (PHP), hypertext markup language (HTML), cascading style sheets (CSS), JavaScript, jQuery, and recent versions of JavaScript libraries as main components for the frontend and MySQL as the database engine. The technologies that have been used for ETE dashboard Web app development include PHP 5.2.4 (minimum), MySQL 5.0.15 or later (MySQL 5.1.x recommended), WordPress, HTML 4 and 5, CSS and CSS3, Google Chart application programming interface (API), scalable vector graphic (SVG) vector (converted from Shapefiles Provided), JavaScript, jQuery, asynchronous JavaScript (Ajax), cURL, and Google Web fonts API. The Web app is compatible with several versions of Google Chrome, Mozilla, Internet Explorer, Safari, Microsoft Edge, and Opera. The minimum Web server requirements include 2 GB disk space/Web space or more, PHP version 5.2.4 or greater, MySQL version 5.0.15 or greater, Apache Server, Apache mod_rewrite module (for clean URIs known as Permalinks), PHP data objects (PDO) database driver for MySQL (pdo_mysql or pdo_mysqli) or Postgres (pdo_pgsql) or MSSQL (pdo_sqlsrv), and PHP Mail Function enabled or simple mail transfer protocol (SMTP) support. The Web app has two main interfaces: frontend interface and admin panel. The frontend is publically accessible, and the admin panel can be accessed by defined username and password assigned to the administrators. The Web app has static and dynamic pages, blog posts, categories, events, charts, graphs, and visualizations, which are manageable from the admin panel. User management includes signup, login, and profile management. The admin panel also manages and filters datasets and creates visualizations.

#### Data Sources for the Dashboard

There are several sources of aggregated data that are utilized and integrated in the dashboard to allow visual exploration of various measures to track and monitor the HIV epidemic.

All data are aggregated from the individual or line level by ETE dashboard staff and then imported into the dashboard. Data sources are shown in Table 1.

#### Data Measures Represented on the Dashboard

The system requires the inclusion and integration of aggregate data from different realms and data sources. Key realms of data include HIV prevention, incidence, testing, new diagnoses, prevalence and care, AIDS diagnoses, and deaths (see Table 2). Key data sources include routine HIV surveillance, vital statistics, eHIVQUAL, Community Health Survey/Behavioral Risk Factor Surveillance System, sexually transmitted infection (STI) surveillance, NYLinks, and Medicaid. A realm can contain information from many data sources (eg, the continuum of care outcome measure, retention in care, is captured both by a facility-reported source, NYLinks, as well as via lab results from HIV surveillance). A data source can likewise contain information that fits into multiple realms.

**Table 1:** Data sources for the Ending the Epidemic (ETE) geovisualization dashboard.

Source	Description (level of data aggregation)	Format	Timeframe	Distribution	Measures
Surveillance data	Population-based registry: electronic reporting of lab tests; physician reports; active field surveillance; case investigations (as low as ZIP code, but may be a larger geographic area when numbers all small to protect privacy)	Prepared in SAS, converted to Excel	Annual	New York State, New York City	Number of new HIV/AIDS^a^ diagnoses, prevalent cases, linkage to care, retention in care, viral load suppression, late and concurrent diagnoses, CD4 count, AIDS diagnoses, deaths among people living with HIV/AIDS
NYC Community Health Survey	Telephone survey (United Hospital Fund neighborhood)	Prepared in SAS, converted to Excel	Annual	New York City (all 5 boroughs)	HIV testing and condom use
Behavioral Risk Factor Surveillance System	Telephone survey (County)	Static reports	Annual	New York State	HIV testing
NY State Department of Health Quality of Care program (eHIVQUAL)	Facility-reported performance measurement review (Facility)	Static reports	Biennial	New York State	Visit frequency, new patient retention, viral load suppression, antiretroviral therapy usage
NYLinks	Facility-reported performance measurement web application (Facility)	Static reports	Bi-monthly, quarterly	Regions: Upper Manhattan, Western New York, Hudson Region, Queens, Long Island	Linkage to care among newly diagnosed, short-term retention, 2-year retention in care
Vital statistics	Registry of all deaths and live births, collected administratively (County)	Excel	Annual	New York State, New York City	Deaths with underlying cause of HIV/AIDS
Medicaid	Administrative billing data (County)	Excel	Annual	New York State	PreP prescriptions filled

^a^AIDS: acquired immune deficiency syndrome.

**Table 2:** Key data measures represented using the Ending the Epidemic (ETE) geovisualization dashboard.

Data realm	Key measures
HIV prevention	Condom use, STI^a^ prevalence, perinatal infections
New infections	Estimated new infections in New York State (NYS) and New York City (NYC), incidence rates
HIV testing	Ever tested for HIV (NYS); tested for HIV in last 12 months (NYC); never tested (NYC)
New diagnoses and linkage	New diagnoses (NYS), HIV diagnosis rates (NYC), median CD4 count at diagnosis, concurrent HIV/AIDS^b^ diagnosis, linkage to care among newly diagnosed within 90 days/30 days
Prevalence and care	HIV/AIDS prevalence, engagement in care, retention in care, antiretroviral therapy use, viral load suppression
AIDS diagnoses	New AIDS diagnoses
Deaths among people with HIV/AIDS	Number of deaths and age-adjusted death rates among people with HIV/AIDS, HIV-related deaths (underlying cause of death HIV/AIDS)

^a^STI: sexually transmitted infection.

^b^AIDS: acquired immune deficiency syndrome.

To meet the ongoing need for routinely updated data, each data source is set up as a stream of aggregate county or ZIP code level data that feed into the various data realms to generate high-level metrics, such as state or city wide trend data on new infections or HIV-related deaths, and additionally allows the user to drill down into specific geographic areas and population groups (eg, by gender, age, or race/ethnicity). Because data in the dashboard are linkable by geography and calendar time, the capability to develop and deploy visualizations that integrate data across multiple realms using data from the dashboard is now possible.

#### Data Integration and Visualization Processes

Data are imported into the ETE in an MS Excel sheet with an .xls or .xlsx extension. They are then converted into Comma Separated Values (CSV) format. Data cleaning is then carried out to remove white spaces and special characters. Lowercase letters and numbers are preferred as variable names in this process. The CSV file is then uploaded to the server and imported into the database by reading it with a PHP script added in a WordPress plugin. After successful import of the datafile, routine checks are carried out to ensure that records were successful saved. The saved records are then converted into JavaScript Object Notation (JSON) content with keys as column titles in first rows. JSON is an open-standard file format that uses human-readable text in transmitting data objects consisting of attribute–value pairs and array data types.

For the visualization process, a custom plugin is created that includes the function, shortcodes, CSS, scripts, images, and SVG files for the visualization functionality. It calls the Google API and loads the scripts. In a separate plugin, a unique shortcode is created for each visualization to be drawn on the frontend. The records of the dataset in question are retrieved in JSON and decoded in the form of PHP arrays. The logic on the PHP array is then applied as per the requirements of visualization (including PHP, JavaScript, jQuery). Data are filtered with JS on the frontend on changing the filter columns. For much larger datasets, the Asynchronous JavaScript and XML (AJAX call) is employed to load the data dynamically on each filter change.

## Methods

### Usage Evaluation of the Dashboard

**Figure 1:**
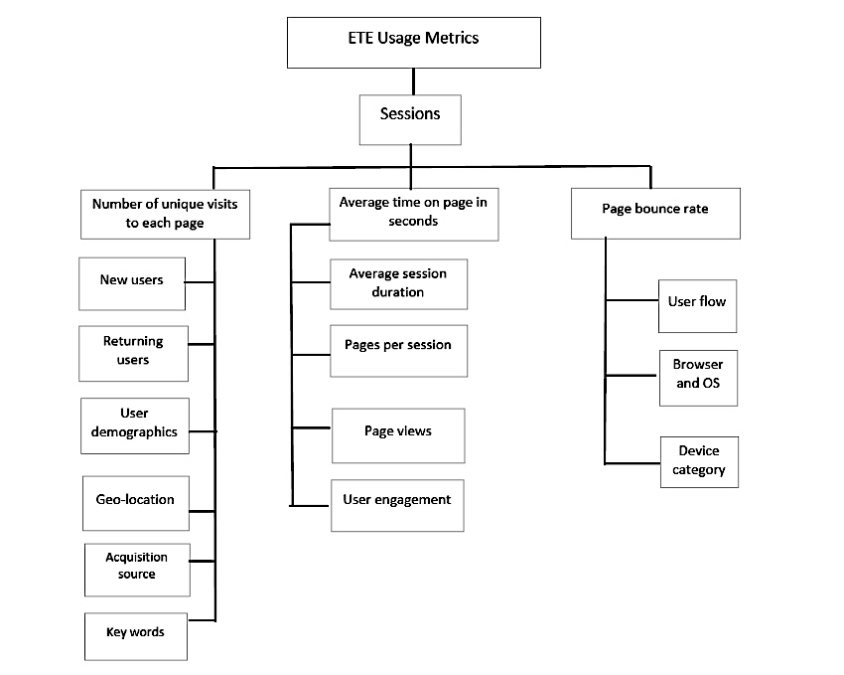
Usage metrics assessed on the ETE dashboard.

Usage evaluation of the ETE geovisualization dashboard was conducted using Google Analytics during a 4-week period from March 19 to April 18, 2016, for which initial analytics data on the static version of the ETE were available (see Figure 1).

Google Analytics reflect real-time views informing the stakeholders of the pages that are most popular and the number of users visiting the website [24]. Prior research has characterized usage based on the following three indicators: (1) number of unique visits to each page, (2) average time on page in seconds, and (3) page bounce rate (ie, percentage of visits where users left the site immediately after viewing only one page). Institutional review board approval was not required for this study since the data used were aggregate data collected for non-research purposes.

### Variables Assessed

The authors assessed the following variables: (1) session, which refers to the period of time in which a user was actively engaged with the website; all usage data are associated with a session, (2) the percentage of new sessions represents the fraction of new sessions initiated by unique users, (3) new and returning users are assessed; new visitors are identified by a unique session, while returning users have previously initiated sessions on the geovisualization dashboard, (4) demographics, which included the age groups and gender of visiting users, (5) bounce rate, which refers to the percentage of single-page visits (ie, visits in which the person left the site from the entrance page without interacting with the page), usually estimated based on a time period, (6) pages per session, which refers to average number of pages viewed during a session (average page depth), (7) average session duration (mins/secs), which refers to the average time spent during a session, (8) unique page views, which refers to the number of sessions during which the specified page was viewed at least once; a unique page view is counted for each page URL + page Title combination, (9) average time on page, which refers to average amount of time users spent viewing a specified page or screen, or set of pages or screens, and (10) average percentage exits, which is estimated as (number of exits) / (number of page views) for the page or set of pages. It indicates how often users exit from that page or set of pages when they view the page(s).

## Results

### Usage Evaluation of the ETE Dashboard

Descriptive analysis of ETE usage was obtained using Google Analytics. Further analysis was also conducted by cross-tabulating specific metrics. The usage metrics generated were classified by acquisition (ie, metrics that define the way users were able to access a given Web portal), behavior (ie, metrics that display user activity on the Web portal), and page visits (ie, characterizing page transitions) (Figure 1).

### Description of Usage Metrics

#### Acquisition

In total, 37.7% of the sessions (324/860) were initiated by unique users (Table 3). The majority of the sessions were initiated across North America (616/860, 71.6%), followed by Asia (191/860, 22.2%) and Europe (51/860, 6%) (Multimedia Appendix 3). The most common acquisition channels included direct source (353/860, 41.0%), followed by referral traffic (340/860, 39.5%), and organic search (134/860, 15.5%). The usage distribution implies the need for improved search engine optimization that facilitates easy location of the ETE site in other parts of the world, since the majority of traffic to the site was via direct sources. Direct traffic represents those users that arrive directly to the site by typing the uniform resource locator (URL) into the browser’s address bar; clicking on a bookmark; or clicking on a link in an email, text message, or chat message. Referral site traffic represents those users who click a link on another site and land on the analyzed site [25]. Organic search refers to users referred by an unpaid search engine listing such as Google.com search [26].

**Table 3:** Usage metrics by acquisition, behavior, and page visits from March 19-April 18, 2016.

Descriptive analysis		Usage metrics
**Acquisition**		
	Sessions, n	860
	New sessions, %	38
**Behavior**		
	New users, n	324
	Returning users, n	536
	Bounce rate, %	46
	Pages per session, n	4.45
	Average session duration, min:sec	8 min:51 sec
**Page visits**		
	Page views, n	3828
	Unique page views, n	1994
	Average time on page, min:sec	2 min:34 sec
	Average % exit for view	22

**Figure 2:**
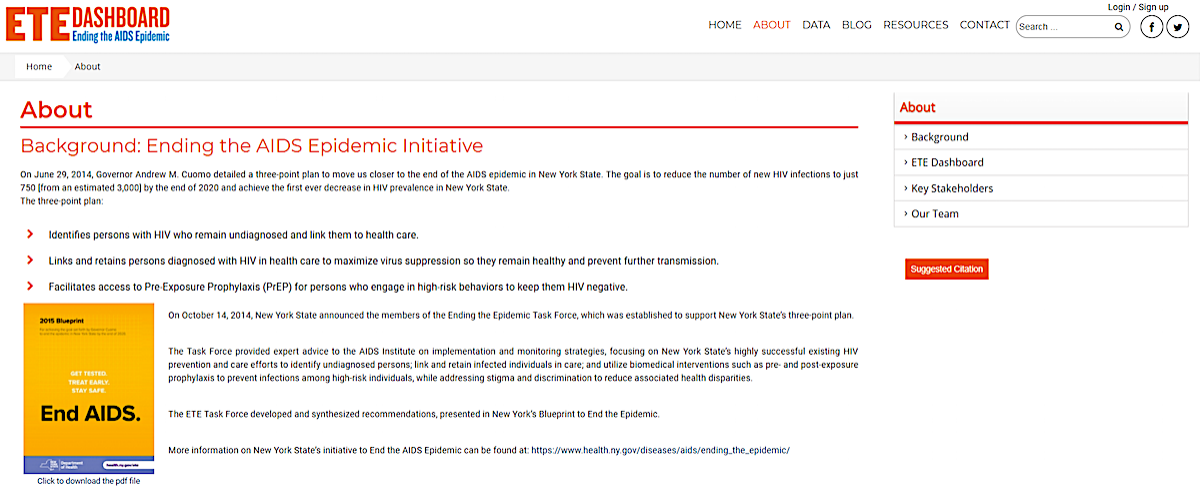
The “About” page of the ETE dashboard.

Of the 528 sessions (N=860 during the time period) where age of users was estimated, more than half were initiated by users within the age group of 25-34 years (320/528, 60.6%) followed by 35-44 years (101/528, 19.1%). Of the 539 sessions in which gender was recorded, the majority were initiated by males (365/539, 67.7%). Of the 136 new users, the proportion of new users being females (78/136, 57.4%) was higher than the males (58/136, 42.6%). Fifteen percent (134/860) of the sessions initiated had records of key words utilized in direct searches. The most common keywords used in accessing the ETE dashboard were not made visible by the Google Analytics platform (115/134, 85.8%). Other common key words included “ete dashboard” (5/134, 4%) and “etedashboardny.org” (4/134, 3%).

### Behavior

The average session duration for both new and returning users was 8 minutes, 51 seconds. Average session duration was higher among returning users (12 min:41 sec), compared to the new users (2 min:31 sec). The total page views for the entire period of observation was 3828, of which 1994 were unique. The average time spent on page viewing was 2 minutes, 34 seconds, and overall page views were highest for the HIV testing page (4 min:1 sec) and lowest for the measures page (1 min:4 sec). Unique page views were highest (413/1194, 34.6%) for the landing page of the ETE dashboard, and users spent an average of 2 minutes, 25 seconds on this page. This page also had one of the highest percentage exits (49.1%, compared to the average bounce rate of 46.3%).

**Figure 3:**
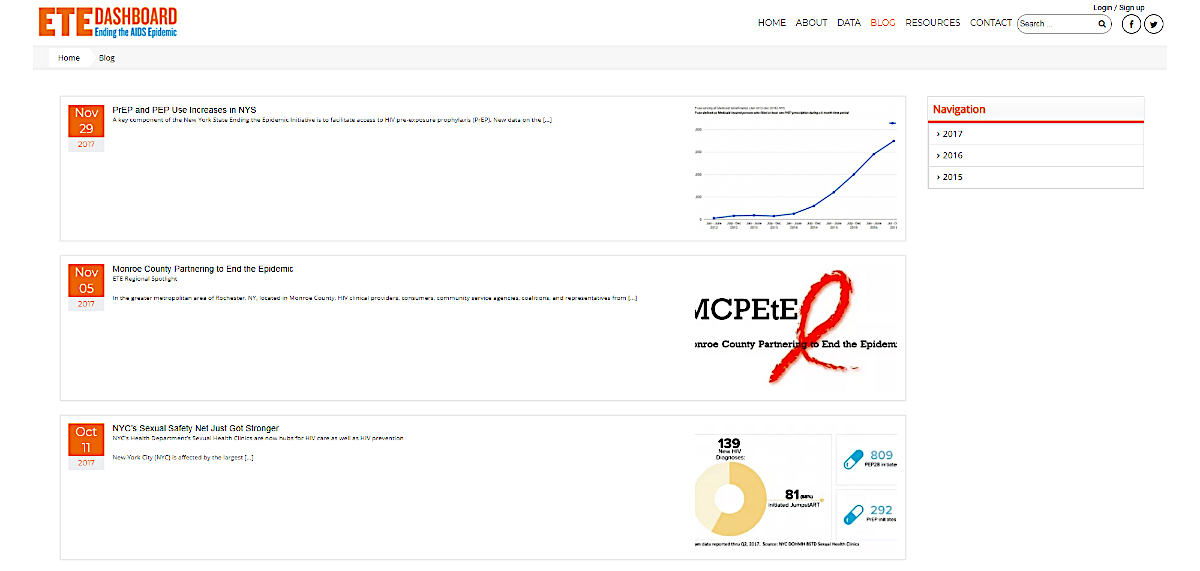
Blog page of the ETE dashboard.

New users (324/860) had a higher average bounce rate of 59.9% compared to returning users (536/860) who had an average bounce rate of 38.1%. The average number of pages viewed per session among the returning users (5.35 pages/session) was also higher compared to the new users (2.35 pages/session). Overall, the average session duration was higher among males (13 min:38 sec) than females (8 min:58 sec). The highest bounce rates were seen for the “about” page, which describes the ETE initiative (bounce rate=65%). The lowest bounce rates were seen for the ETE blog page (bounce rate=17%). See Figures 2 and 3.

**Figure 4:**
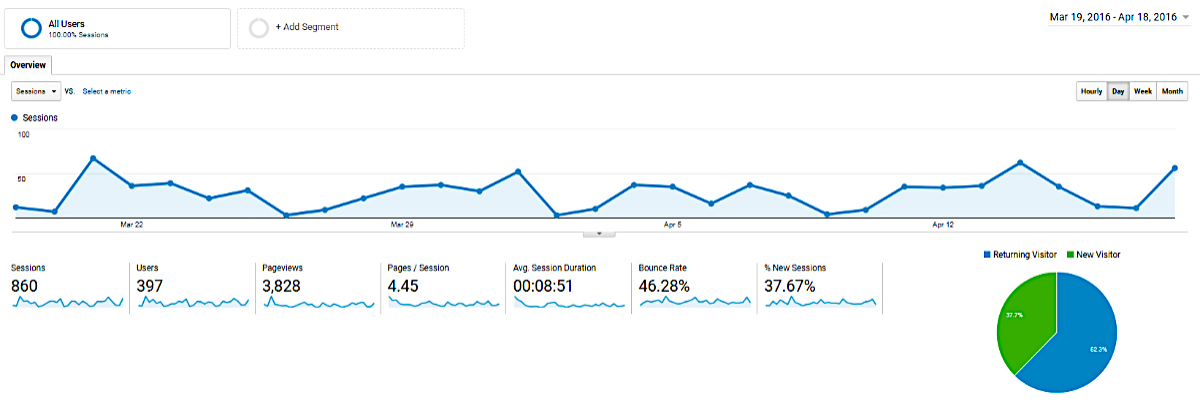
Usage patterns from March 19-April 18, 2016 (Google Analytics screenshot).

### Browser Operating System and Device Category

Google Chrome was the most common Web browser chosen by users in accessing the ETE geovisualization dashboard (459/860, 53.4%). This was followed by Internet Explorer (194/860, 22.6%), Mozilla Firefox (108/860, 12.6%), and Safari (87/860, 10.1%). The average bounce rate across the browsers was 46.3%, and this was highest among those using Mozilla Firefox (59.3%) and least for Google Chrome users (37.0%).

Bounce rate was similar for Safari (54.0%) and Internet Explorer users (55.2%). Desktops were the most common devices used in accessing the ETE dashboard (820/860, 95.3%). Other devices included mobile phones (37/860, 4%) and tablets (3/860, 0.4%). Bounce rates were highest among tablet users (66.7%) with an average session duration of 1 minute, 19 seconds. However, the number of pages viewed per session was similar between tablet users (4.00) and desktop users (4.56). Mobile device users had the lowest page per session views (2.14).

#### Page Visits

The user flow describes the overall page path level including the landing pages, transition pages, and exit pages accessed by the user. The landing page with the maximum page views was the ETE home page, which is a static page that provides a snapshot of estimated metrics for tracking progress towards ending the HIV epidemic in New York. The home page also outlines the initiative’s objectives and key stakeholders. Following the home page, user traffic on the ETE dashboard was maximum on the HIV Care Cascades page, which is a dynamic page that allows users to self-select different cascades of HIV care by various data measures. The top exit page through which users left the ETE dashboard was the home page. Bounce rates were slightly higher for the static home page (49.4%) compared to the dynamic cascade page (47.1%). Users also spent more time on the HIV Care Cascades page (15 min:16 sec) (Multimedia Appendix 4).

#### Usage Patterns

Figure 4 shows the variation in usage patterns during the 4-week period from March 19 to April 18, 2016, for which initial analytics data on the static version of the ETE were available. Site usage was lowest on April 2, with a total of 3 sessions initiated, and highest on March 21 with a total of 67 sessions initiated. In particular, the least site usage (exemplified by lowest points on the chart below) was observed during weekends (Saturdays and Sundays). New data were ported into ETE during the time period of assessment, but only for display on the test site and not the public URL.

## Discussion

### Principal Findings

This study describes the design and usage analysis of the ETE geovisualization dashboard, with the aim of integrating epidemiologic and other data and disseminating knowledge on the HIV epidemic in New York State in the context of the Ending the Epidemic Initiative. Empirical reports indicate that regardless of the timeliness and accuracy of disease surveillance data collection, translation of data into accessible and useful knowledge will ultimately determine its utility in decision making [27]. Hence, the modality for disseminating aggregate data is crucial. The relevance of visualization tools and technologies is underscored by their ability to explore the relationships between data elements beyond the capacity of traditional statistical analysis in a timely manner, thereby providing an effective tool for informing decision making by health system planners, policy makers, and other key stakeholders. The unique benefits of visualization tools in reducing cognitive effort in data analysis allows users with limited expertise in data visualization or statistical analysis to effectively and rapidly gain key insights. In addition, geovisualization technologies provide a user-friendly interface that enhances user experience and enables seamless visualization of complex data. A distinguishing feature of the ETE dashboard is its flexible architecture adapted from SanaViz, which allows the incorporation of additional features and functionalities that enhance user experience in disease monitoring and assessment. Presently, the ETE dashboard is undergoing further development to enhance user interactivity and maximize data translation across all of its key data sources and realms.

Commercial use of Google Analytics has been promising [28]. Google Analytics has been widely used in examining usage metrics and predicting progress and challenges of a variety of surveillance-based and Internet-delivered eHealth interventions including HIV monitoring, mental health, neurosurgery, depression, stress, and anxiety [29-32]. Our findings reveal the potential of Google Analytics as a tool to enhance user traffic and performance of the ETE geovisualization dashboard by identifying key user metrics for potential modification. Usage statistics of the ETE geovisualization dashboard indicate that for the 860 sessions initiated by both new and returning users, the average session duration was 8 minutes, 51 seconds, and the bounce rate was 46%. These statistics reflect positive results given that prior literature estimates a bounce rate of 40-60% for most websites. Prior studies examining Internet-delivered interventions report an average range of site visit durations between 7-13 minutes [29,33]. However, the average viewing time for the ETE was 2 minutes, 31 seconds, reflecting lower usability, since prior studies assessing website performance have reported an average page viewing time exceeding 3 minutes as an indicator of good usability [13]. Similarly, our results showed a range of page visits between 1 and 12 pages per session, possibly indicative of content issues since previous studies report page visits between 3 and 17 pages as markers of interest in page content [13].

Key findings from our study showed that the highest bounce rates were seen for the “about” page, which describes the ETE initiatives (65%). The lowest bounce rates were seen for the ETE blog page (17%), which is consistent with prior research on website usage metrics using Google Analytics, which indicates that blog pages are often the most frequently viewed pages on Web portals [33]. The ETE blog page in particular is the most frequently updated page on the ETE system, providing research, news, and updates on a routine basis. Blogs pages are of increasing interest to users and have recorded lower bounce rates compared to the majority of website pages [33]. This has been attributed to various factors including their ability to serve as a medium for conveying advice to policy makers and the idea that such policy blogs were written by credible stakeholders [33]. However, more recent studies focused on engaging policy makers with research findings report that the number of articles, and not blogs, may be a more significant predictor of webpage visits and sustained usage and may be attributed to topic relevance [34]. In addition, page views for blogs were significantly higher for those targeted at one’s agency compared to external agencies, with a stronger association for internally authored blogs [34]. This indicates a variety of considerations that need to be accounted for in trying to drive usage for specific pages. Further analysis will be required over a longer time period to better capture the user demographics for the ETE site.

More than two-thirds of the sessions were initiated in North America (72%) indicating the need for improved search engine optimization that facilitates easy location of the ETE site in other parts of the world. Search engine optimization is relevant especially since most of the traffic arriving at the ETE site was via direct traffic (41%), which is consistent with prior studies indicating direct traffic as one of the highest acquisition channels [35].

Desktop computers were the most common devices used in accessing the ETE geovisualization dashboard (95%), compared to mobile phones (4%) and tablets (0.4%). This may suggest the need for better optimization of the ETE dashboard on mobile and tablet platforms. Prior research has shown that websites designed for desktop usage and not optimized for mobile usage may hamper incoming traffic [36]. In addition, mobile analytics is yet to be fully explored, as data collection using mobile devices has not been fully optimized [36]. Google Chrome was the predominant browser chosen by users in accessing the dashboard (53%) among others, including Internet Explorer (23%), Mozilla Firefox (13%), and Safari (10%).

### Strengths and Limitations

A strength of this study is its description of the utilization and significance of interactive dashboards in disseminating information related to public health programs and policies to diverse stakeholders for better informed decision making. However, a limitation is that the usage evaluation of the ETE dashboard was conducted for short period and it may require evaluation for a longer period.

### Future Work

Future studies will employ a user survey among stakeholders of the ETE program to adequately characterize usage patterns and further evaluate user-friendliness and interactivity of the ETE geovisualization dashboard from the perspectives of potential users, in line with the human-centered design approach [37].

### Conclusion

Enhancing user-friendliness of a system provides added benefits of user satisfaction, acceptability, alongside financial benefits such as improved product quality and reduced production and support costs [37]. The data gathered from the usability assessment of the ETE will be used to further modify the ETE dashboard for optimal performance and utility.

## Appendix

Multimedia Appendix 1 Static version of the ETE dashboard.

Multimedia Appendix 2 Interactive version of the ETE dashboard.

Multimedia Appendix 3 Usage distribution of the ETE dashboard by geolocation from March 19-April 18, 2016.

Multimedia Appendix 4 User transitions across the ETE dashboard.

## Abbreviations

AIDS: acquired immune deficiency syndrome
AJAX: asynchronous JavaScript
API: application programming interface
CSS: cascading style sheets
CSV: Comma Separated Values
eHIVQUAL: New York State Department of Health Quality of Care program
ETE: Ending the Epidemic
HTML: hypertext markup language
JSON: JavaScript Object Notation
NYC: New York City
NYS: New York State
PHP: Hypertext Preprocessor
SQL: structured query language
SVG: scalable vector graphic
