# Supplementary material for: A Human-Centered Platform for HIV Infection Reduction in New York: Development and Usage Analysis of the Ending the Epidemic (ETE) Dashboard
Source: JMIR Public Health Surveill. 2017 Dec 11;3(4):e95. doi: 10.2196/publichealth.8312 (PMC5742657; doi:10.2196/publichealth.8312)
Supplement: Multimedia Appendix 2 [file publichealth_v3i4e95_app2.pdf]

HIV TESTING NYC

34% NYC residents tested in last 12 months (2013)

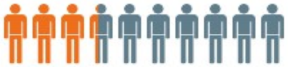

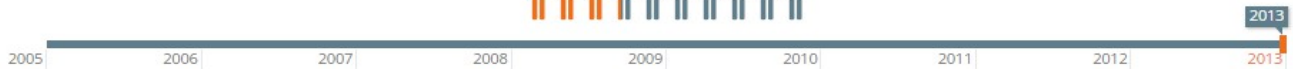

Select HIV testing indicator:

- (•) Tested in last 12 months
- ( ) Never Tested
- ( ) Tested among MSM
- ( ) Never Tested among MSM

Filters

SEX: Total

RACE: Total

AGE: Total

Reset Filters

Highlight By Location

Search UHF name and zip code

West Queens
2013

Select an area on the map to view UHF neighborhood level data
Tested in last 12 months, 2013

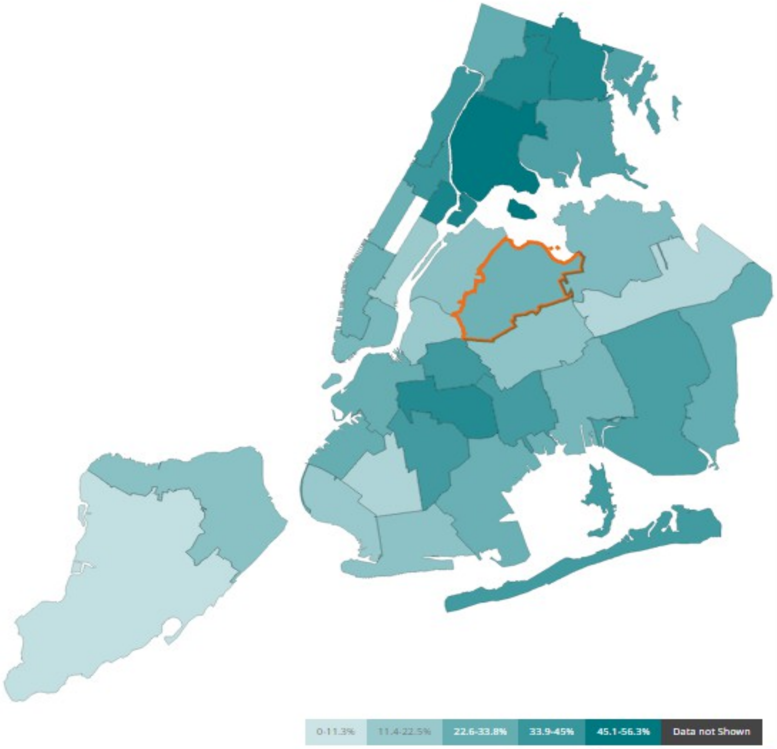

Reset UHF Selection

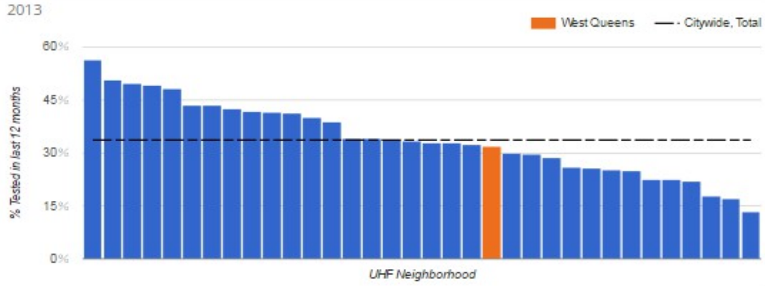

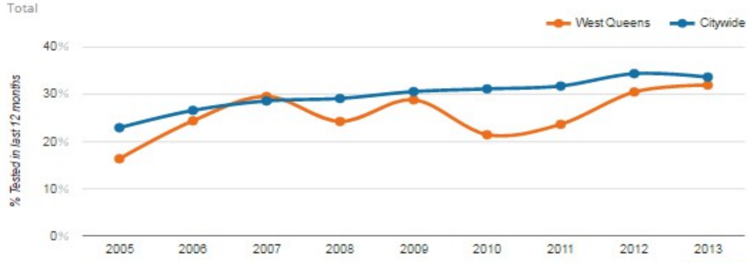

Show 95% Confidence Intervals OFF

Data Source: NYC Community Health Survey
All estimates are age-adjusted to the year 2000 U.S. Census
