# Supplementary figures and images for: A Human-Centered Platform for HIV Infection Reduction in New York: Development and Usage Analysis of the Ending the Epidemic (ETE) Dashboard
Source: JMIR Public Health Surveill. 2017 Dec 11;3(4):e95. doi: 10.2196/publichealth.8312 (PMC5742657; doi:10.2196/publichealth.8312)

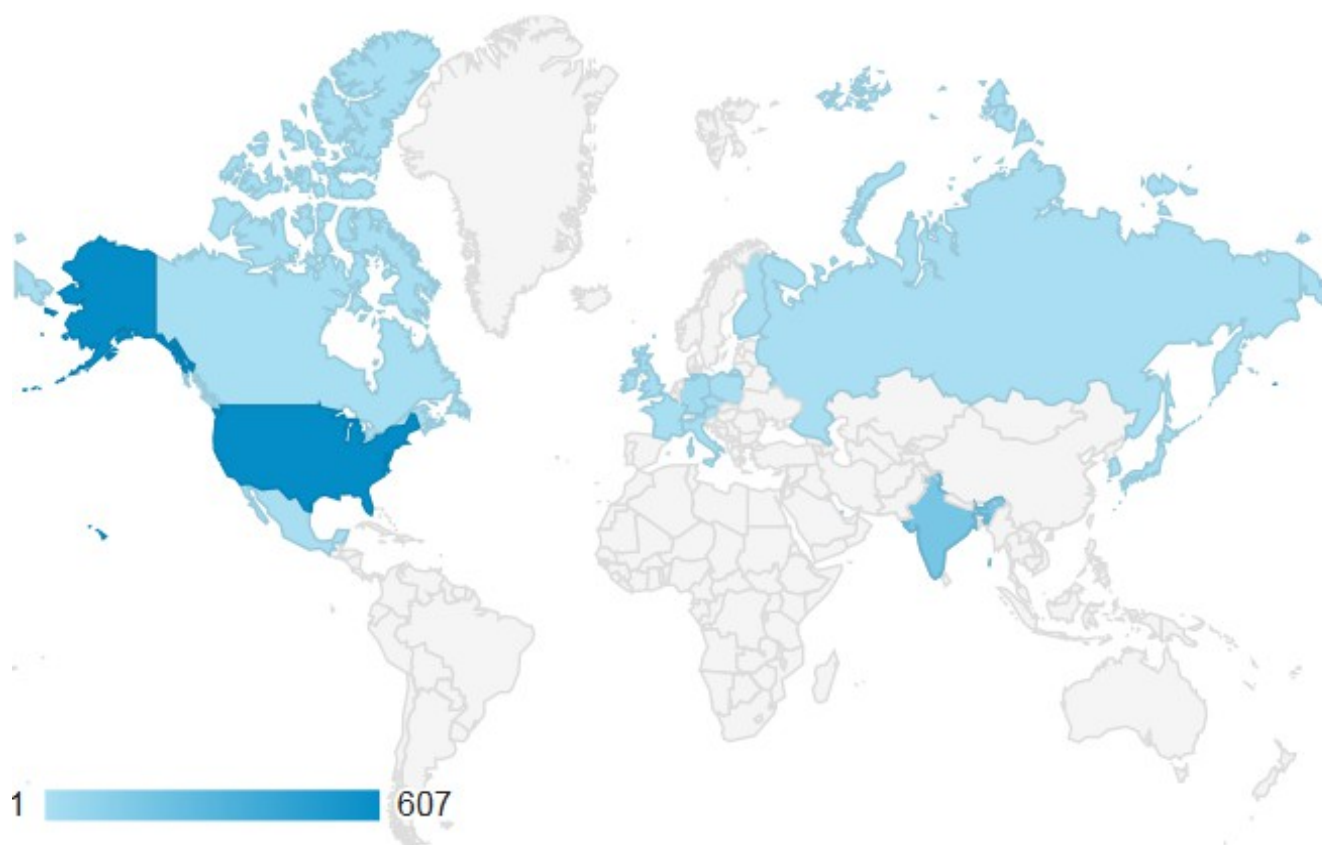

Supplement: Multimedia Appendix 3 [file publichealth_v3i4e95_app3.pdf]
