# Supplementary material for: A Human-Centered Platform for HIV Infection Reduction in New York: Development and Usage Analysis of the Ending the Epidemic (ETE) Dashboard
Source: JMIR Public Health Surveill. 2017 Dec 11;3(4):e95. doi: 10.2196/publichealth.8312 (PMC5742657; doi:10.2196/publichealth.8312)
Supplement: Multimedia Appendix 4 [file publichealth_v3i4e95_app4.pdf]

Top landing page

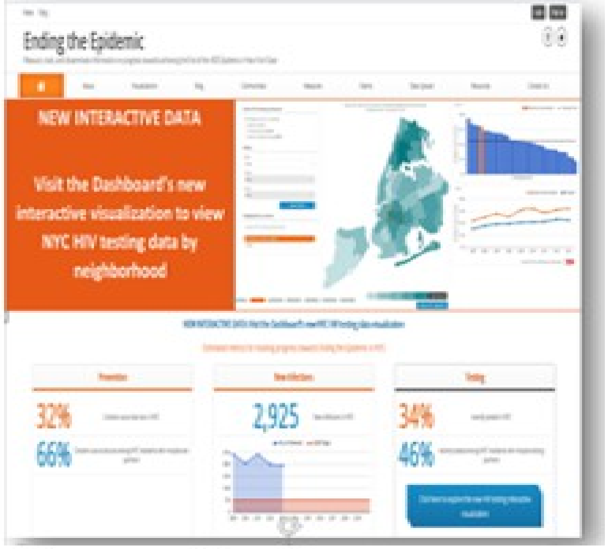

Page source: "Home"
Page link: http://etedashboardny.org/
Page bounce rate: 49%
Average duration on page:3mins 26secs

Top transition page

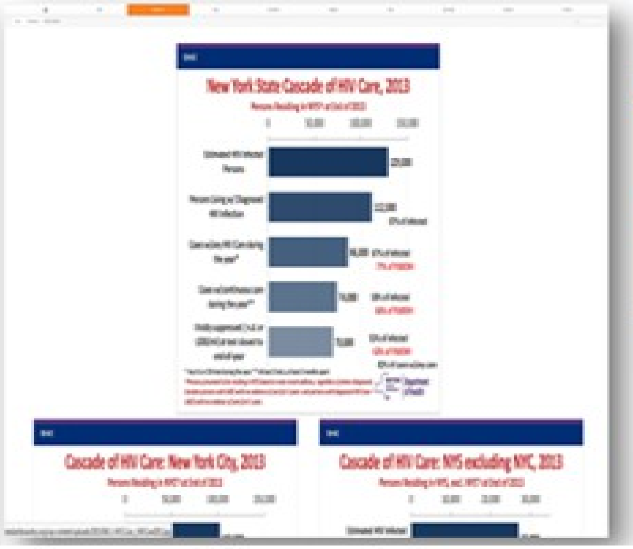

Page source: "HIV care cascades"
Page link:
http://etedashboardny.org/visualizations/hiv-care-cascades/
Page bounce rate: 47%
Average duration on page:15mins 16secs

Top exit page

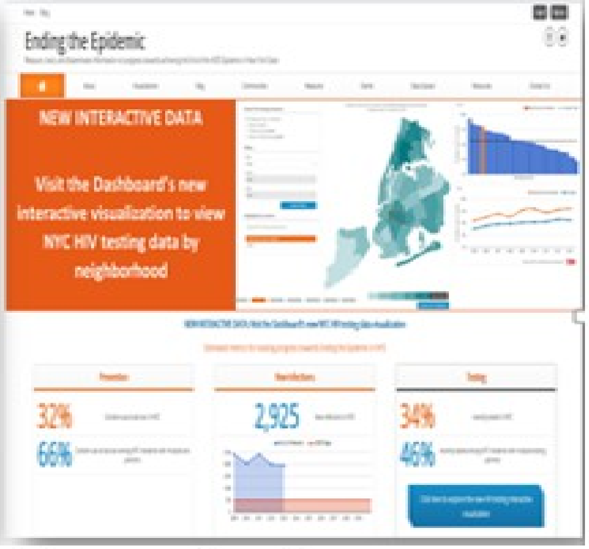

Page source: "Home"
Page link: http://etedashboardny.org/
Page bounce rate: 49%
Average duration on page:3mins 26secs
